# Aflatoxin B1, ochratoxin A, and fumonisin B1 detoxification from poultry feeds by corona discharge application

**DOI:** 10.5455/javar.2024.k834

**Published:** 2024-12-26

**Authors:** Hiba S. Alnaemi, Tamara N. Dawood, Qais Th. Algwari

**Affiliations:** 1 Department of Veterinary Public Health, College of Veterinary Medicine, University of Mosul, Mosul, Iraq; 2 Department of Veterinary Public Health, College of Veterinary Medicine, University of Baghdad, Baghdad, Iraq; 3 Department of Electronics, College of Electronic Engineering, University of Ninevah, Mosul, Iraq

**Keywords:** Aflatoxin B1, corona discharge, feeds, fumonisin B1, ochratoxin A

## Abstract

**Objective:**

The efficiency of corona discharge (CD) for detoxification of aflatoxin B1 (AB1), ochratoxin A (OA), and fumonisin B1 (FMB1) from poultry feeds with its influences on feed components was investigated.

**Materials and Methods:**

Feed samples were exposed to CD for six durations (10, 20, 30, 40, 50, and 60 min) at three distances (1.5, 2.5, and 3.5 cm). Mycotoxin levels were estimated by competitive enzyme-linked immunosorbent assay, and findings were substantiated by high-performance liquid chromatography.

**Results:**

AB1, OA, and FMB1 degradation percentages increased significantly (*p* < 0.05) with processing times increment and distances reduction to reach values of 83.22%, 84.21%, and 84.76% at the first distance; 80.28%, 84.00%, and 84.12% at the second distance; and 68.30%, 71.74%, and 76.18% at the third distance, respectively, after 60 min of treatment. FMB1 reported the highest degradation level. Concerning CD impacts on feed composition, protein, fat, and moisture contents decreased significantly (*p* < 0.05). Carbohydrates and ash were not affected adversely. Depending on peroxide values estimation, fats were of good quality.

**Conclusion:**

The CD effectiveness for AB1, OA, and FMB1 detox from poultry feeds with moderate impact on the quality of feed.

## Introduction

Mycotoxins are secondary metabolites created by mycotoxigenic molds that have multiple toxicological influences on humans and animals. Several mycotoxins have been specified, but the consistently confronted mycotoxins that constitute an apprehension for the health of humans and animals and have economic importance are aflatoxins, ochratoxins, and fumonisins [1–3]. Mycotoxigenic molds are present around the globe, resulting in the appearance of mycotoxins in the food series [4–7]. Exposure of humans to mycotoxins can take place directly and indirectly through the consumption of contaminated foods derived from plants and animals, respectively. AB1, OA, and FMB1 are classified as the most serious mycotoxins due to their hepatotoxic, immunotoxic, nephrotoxic, neurotoxic, mutagenic, carcinogenic, and teratogenic influences [1, 8].

As mycotoxins comprise an immense health menace to humans and animals and result in enormous economic losses, for this cause, approaches to eradicate or deactivate mycotoxins in foods and feeds are imperiously required. These approaches can be assorted into three fundamental divisions: physical, chemical, and biological approaches [9–11]. Non-thermal physical approaches came into light in the last few decades due to their low cost, low time consumption, low undesirable effects on the food matrix, the ability to maintain good sensory and textural food characteristics, and the capability to use them with all kinds of foods and feeds [12]. Cold plasma (CP) is classified as one of the most contemporary and promising approaches employed to reduce the levels of mycotoxins in foods and feeds significantly. The most significant degradation influence of CP on mycotoxins is the rapid reaction of the created reactive species (RS), which include electrons, radicals, and reactive oxygen and nitrogen species, with double and triple bonds, functional groups, and various effective rings linked to the structure of mycotoxins. Mycotoxin destruction leads to the formation of less or non-toxic compounds compared to the original mycotoxin [13]. Mycotoxin degradation using different forms of cold plasma was demonstrated by several studies [14–16].

Corona discharge (CD) in the air at atmospheric pressure is one of the simplest provenances of CP, known to produce chemically RS acting as powerful oxidizers [16]. Investigation of CD efficiency for mycotoxin degradation in feed was not documented. Corona discharge plasma jet (CDPJ) for AFB1 degradation was only inspected by Puligundla et al. [16] on glass slides and in artificially spiked rice and wheat. Therefore, the study aimed to detect the effectiveness of CD in AB1, OA, and FMB1 degradation in the feeds of poultry that were naturally contaminated and to establish the effective parameters for their degradation, in addition to evaluating the impact of CD on the feed nutritional ingredients and peroxide values (PVs).

## Materials and Methods

### Samples of feed

Samples of poultry feeds (pelleted type) were gained from factories of feeds and farms of poultry in Ninevah Governorate, Iraq, during the period from April to September of the year 2022. After sample homogenization, 1 kg of representative samples was obtained, milled, and sieved (sieve with mesh NO. 18). The samples that demonstrated AB1, OA, and FMB1 co-occurrence at the greatest values were utilized for CD exposure.

### CD generation and application on feed samples

The CD system comprised several components, including a high-voltage source as the main part. High-voltage production is achieved by boosting a direct current (DC) via a zero-voltage switch circuit with a transformer. Since the electrodes are an essential part of the CD system, therefore, two copper electrodes are assembled around the capillary tube. For gas injection, an air pump was used to feed the discharge system with working gas. Finally, the sample exposure plate was used for treatment. Plasma was generated under normal atmospheric pressure using a 24 V DC power source. The output voltage reached approximately 15 kV with a frequency of 100 Hz. The plasma, produced via corona discharge between stainless steel electrodes, exhibited streamer-like properties. To produce a plasma jet or plume, a Model RS-510 air pump (Bulgaria) was used, providing a constant flow rate of 2.5 l per minute (lpm) with dry air serving as the working gas or carrier gas. Ten grams of naturally co-contaminated feed samples were put in a translucent polypropylene box (21.5 × 18 × 4 cm) that was modified to permit plasma generation. The samples were placed in a superfine layer of approximately 0.1 cm and exposed to CD for six durations (10, 20, 30, 40, 50, and 60 min) at three electrode-to-sample plate distances (1.5, 2.5, and 3.5 cm). The samples were stirred every 5 min, and each combination of time and distance was tested in triplicate. As the air was used as a working gas, O_3_, nitric oxide (NO), and nitrogen oxides (NO_x_) were estimated as indicators for ROS and RNS generated during CD application, respectively, in addition to sample temperature measurement during different time durations and distances. Ozone concentration was estimated using a portable O_3_ gas detector at 100 ppm, in China. NO and NO_x_ were estimated using a gas analyzer (TESTO 330-2 LL, Germany). Sample temperature was measured using a digital thermometer.

### AB1, OA, and FMB1 quantification in feeds of poultry by enzyme-linked immunosorbent assay (ELISA)

AB1, OA, and FMB1 levels in samples of feeds before and after CD treatment were determined by ELISA kits (Elabscience Biotechnology Inc., USA). Feed samples pretreatment and procedures of competitive ELISA for detection of AB1, OA, and FMB1 were executed by the instructions of the kit producer. The optical density amounts were calculated by a reader of a microtiter plate (HumaReader HS, Germany).

### Substantiation of results by HPLC analysis

HPLC analysis was achieved on the samples of poultry feeds that exhibited a simultaneous occurrence of AB1, OA, and FMB1 at the greatest concentrations for CD exposure. Likewise, samples that manifested the greatest degradation values of mycotoxins after non-thermal exposure were examined by HPLC.

HPLC examination was conducted for AB1 in accordance with [17], OA in accordance with [18], and FMB1 by [19]. The standards of AB1, OA, and FMB1 were gained from Sigma-Aldrich (USA). Sample clean-up was executed utilizing SEP-PAK^® ^silica cartridges (Waters Corporation, USA) for AB1, SEP-PAK^®^ C18 cartridges (Waters Corporation, USA) for OA, and cartridges for strong anion extraction (SAX) (InertSep SAX, GL Sciences, Japan) for FMB1. Mycotoxin determination was accomplished using an HPLC with a fluorescence detector from Shimadzu Corp., Japan. A NUCLEODUR^®^ C18 column (4.6 × 250 mm, Germany) was employed.

### Percentages and kinetics of mycotoxin degradation

Percentages of AB1, OA, and FMB1 degradation after CD exposure were estimated in accordance with the equation:

Mycotoxin degradation (%) = (1−C_t_/C_0_) × 100 

where

C_t_ = AB1, OA, and FMB1 concentrations at time (t).

C_0_ = AB1, OA, and FMB1 initial concentrations at zero time.

The kinetics of mycotoxin degradation were modeled utilizing the exponential decay model [14], which can be fit based on the equation:

y = y_0_+A_1_*exp (-(x-x_0_)/t_1_)

Where:

y ≡ C_t_; y_0_ ≡ C_0_; x ≡ t; A_1_, x_0_ and t_1_ are constants.

### Assessment of the CD influence on the nutritional components and peroxide values of the feeds

Moisture in feeds depending upon water forfeit-on-drying at a temperature of 135°C for 2 h was measured by the method of AOAC 930.15 [20]. Total carbohydrate was valued according to the phenol-sulfuric acid procedure [21]. Determination of crude fat was done utilizing a Soxhlet apparatus according to the method 920.39 [22]. Estimation of crude protein was performed employing the Kjeldahl method as per method 984.13 of AOAC [23]. The method of AOAC 942.05 was espoused for ash estimation [24]. Method 965.33 counseled by AOAC [25] was embraced to determine the peroxide values of the extracted fats.

### Statistical analysis

Statistical analysis of the data was accomplished utilizing the Two-Way Analysis of Variance of the Sigma Stat Version 3.10. A comparison of the means was carried out by Duncan's Multiple Range Test at *p *< 0.05 [26].

## Results and discussion

### RS of CD and sample temperature

Results concerning the detection of RS produced during corona discharge exposure exhibited the predominance of ozone over the other estimated RS. Ozone levels increased significantly (*p* < 0.05) with processing times increment and electrode-to-sample plate distances reduction, to record amounts of 24.5, 22.41, and 19.01 parts per million (ppm) after 60 min from corona discharge exposure at the first, second, and third distances, respectively (Table 1).

**Table 1.** Ozone gas mean concentrations during CD application on feed samples at different time durations and distances.

Time (min)	Ozone concentration (ppm)			
	1.5 cm	2.5 cm	3.5 cm	Time effect
0	0 ^a^	0 ^a^	0 ^a^	0 ^A^
2	11.83 ^b^	10 ^c^	6 ^d^	9.28 ^B^
4	16.26 ^e^	14.2 ^f^	9.04 ^g^	13.17 C
6	17.63 ^h^	14.85 ^i^	11.6 ^j^	14.69 ^D^
8	19.77 ^k^	16 ^l^	13.98 ^m^	16.58 ^E^
10	20.75 ^n^	17.89 ^o^	15 ^p^	17.88 ^F^
20	22.53 ^q^	20.24 ^r^	17.07 ^s^	19.95 ^G^
30	23.78 ^t^	21.84 ^u^	18.58 ^v^	21.4 ^H^
40	24.2 ^w^	22.02 ^x^	19 ^v^	21.74 ^I^
50	24.5 ^w^	22.38 ^x^	19.03 ^v^	21.97 ^I^
60	24.5 ^w^	22.41 ^x^	19.01 ^v^	21.97 ^I^
Distance effect	18.70 ^A^	16.53 ^B^	13.48 ^C^	

Vertically different small letters are significantly different at (*p* < 0.05), horizontally different small letters are significantly different at (*p* < 0.05), and different capital letters within the last row and column are significantly different (*p* < 0.05).

Perceptible values of NO and Nox (1 ppm) were recorded after 5 min at the first distance and 10 min at the second and third distances and continued until 60 min of CD application. Chang et al. [27] exhibited identical results when they found the sovereignty of ozone over the other assessed RS during exposure to corona discharge.

During CD treatment, sample temperature increased slightly to 25.4°C, 23.4°C, and 22.5°C after 60 min from the application of CD at distances of 1.5, 2.5, and 3.5 cm, respectively, compared with the temperature recorded at zero time (20.1°C). This increment was extremely little and inadequate to participate in mycotoxin degradation. A similar finding was indicated by [15], where the sample temperature reached 40°C after CP application.

### Degradation of AB1, OA, and FMB1 after corona discharge exposure

A linear decline in AB1, OA, and FMB1 levels in the samples of poultry feeds after treatment with CD was reported with a significant increment (*p* < 0.05) in examined mycotoxins degradation percentages to record values of 83.22%, 84.21%, and 84.76% at the first distance; 80.28%, 84.00%, and 84.12% at the second distance; and 68.30%, 71.74%, and 76.18% at the third distance, respectively, after 60 min. Results illustrated that exposure to CD for 10 min was enough to reduce AB1 levels at the three studied distances to levels less than the regulatory limit for AB1 (20 µg/kg) established by the European Commission (EC) in poultry feeds [28]. Likewise, 10 min of exposure to CD at the first and second distances was sufficient to reach the EC regulatory limit for OA in poultry feeds (100 µg/kg) [29], while 20 min at the third distance was imperative to achieve the legal limit. Concerning FMB1, feed samples employed for CD exposure that exhibited the greatest concentrations were within the permissible level for FMB1 + FMB2 in feeds of poultry (20,000 µg/kg), where no allowable limit was established by the EC appertaining to FMB1 alone [29] (Tables 2–4).

Results illustrated significant influences (*p* < 0.05) for each of the exposure times and the electrode-to-sample plate distances on AB1, OA, and FMB1 degradation competence during the application of corona discharge that increased with increasing the times of exposure and decreasing the electrode-to-sample distances, except the degradation percentages of AB1 that revealed insignificant differences (*p* < 0.05) at the first and second distances (Tables 5–7).

Studies exploring the degradative influences of corona discharge on mycotoxins are rare. CDPJ efficacy in AB1 degradation in artificially contaminated rice and wheat was studied by [16], who found a significant reduction (*p* < 0.05) in levels of AB1 with application times increment to register 56.6% and 45.7% reduction levels, respectively, after exposure to 30 min at 15 mm distance. In the current study, the degradation percentage of AB1 (65.49%) after exposure to the same time duration and distance was greater than the percentages reported by [16], which may be ascribed to the great surface area that is liable to the deterioration influences of the generated RS as a result of subjecting the milled feed to CD processing.

**Table 2.** Effect of CD at 1.5 cm on AB1, OA, and FMB1 mean concentrations and degradation percentages in poultry feeds.

Treatment time (min)	AB1		OA		FMB1		Time effect
	Conc. (µg/kg)	Deg. (%)	Conc. (µg/kg)	Deg. (%)	Conc. (µg/kg)	Deg. (%)	
0	23.71	0 ^a^	164.66	0 ^a^	6,850.25	0 ^a^	0 ^A^
10	12.62	46.62 ^b^	82.24	49.83 ^b^	2,834.07	58.63 ^c^	51.69 ^B^
20	10.01	57.73 ^d^	50.9	68.93 ^e^	1,667.52	75.66 ^f^	67.44 ^C^
30	8.17	65.49 ^g^	42.81	73.91 ^h^	1,481.91	78.37 ^fl^	72.59 ^D^
40	6.95	70.59 ^i^	37.17	77.38 ^h^	1,356.11	80.20 ^hl^	76.06 ^E^
50	5.29	77.74 ^j^	29.35	82.08 ^k^	1,118.60	83.67 ^kh^	81.16 ^F^
60	3.98	83.22 ^k^	25.9	84.21 ^k^	1,044.21	84.76 ^k^	84.06 ^G^
Mycotoxin effect		57.34 ^A^		62.33 ^B^		65.9 ^C^	

Vertically different small letters are significantly different at (*p* < 0.05), horizontally different small letters are significantly different at (*p* < 0.05), and different capital letters within the last row and column are significantly different (*p* < 0.05), concentration (Conc.), degradation (Deg.).

**Table 3.** Effect of CD at 2.5 cm on AB1, OA, and FMB1 mean concentrations and degradation percentages in poultry feeds.

Treatment time (min)	AB1		OA		FMB1		Time effect
	Conc. (µg/kg)	Deg. (%)	Conc. (µg/kg)	Deg. (%)	Conc. (µg/kg)	Deg. (%)	
0	23.71	0 ^a^	164.66	0 ^a^	6,850.25	0 ^a^	0 ^A^
10	12.30	48.04 ^b^	89.33	45.54 ^b^	2,993.23	56.30 ^c^	49.96 ^B^
20	10.66	55.07 ^d^	64.73	60.55 ^e^	2,253.02	67.11 ^f^	60.91 ^C^
30	9.40	60.38 ^g^	52.46	68.01 ^h^	2,022.92	70.47 ^hf^	66.29 ^D^
40	7.90	66.65 ^i^	41.87	74.49 ^j^	1,581.76	76.91 ^j^	72.68 ^E^
50	6.26	73.68 ^k^	31.51	80.79 ^l^	1,241.93	81.87 ^l^	78.78 ^F^
60	4.66	80.28 ^l^	26.21	84.00 ^l^	1,087.7^1^	84.12 ^l^	82.8 ^G^
Mycotoxin effect		54.87 ^A^		59.05 ^B^		62.4 ^C^	

Vertically different small letters are significantly different at (*p* < 0.05), horizontally different small letters are significantly different at (*p* < 0.05), and different capital letters within the last row and column are significantly different (*p* < 0.05), concentration (Conc.), degradation (Deg.).

**Table 4.** Effect of CD at 3.5 cm on AB1, OA, and FMB1 mean concentrations and degradation percentages in poultry feeds.

Treatment time (min)	AB1		OA		FMB1		Time effect
	Conc. (µg/kg)	Deg. (%)	Conc. (µg/kg)	Deg. (%)	Conc. (µg/kg)	Deg. (%)	
0	23.71	0 ^a^	164.66	0 ^a^	6,850.25	0 ^a^	0 ^A^
10	16.84	28.92 ^b^	114.54	30.17 ^b^	4,320.67	36.93 ^c^	32.01 ^B^
20	14.31	39.44 ^d^	97	40.91 ^d^	3,749.26	45.27 ^d^	41.87 ^C^
30	12.20	48.45 ^e^	87.39	46.74 ^e^	3,011.52	56.04 ^f^	50.41 ^D^
40	10.46	55.88 ^g^	69.04	57.92 ^g^	2,476.69	63.84 ^h^	59.22 ^E^
50	9.41	60.27 ^gh^	56.9	65.37 ^h^	1,877.84	72.59 ^i^	66.08 ^F^
60	7.50	68.30 ^j^	46.35	71.74 ^jk^	1,631.44	76.18 ^ki^	72.07 ^G^
Mycotoxin effect		43.04 ^A^		44.69 ^A^		50.12 ^B^	

Vertically different small letters are significantly different at (*p* < 0.05), horizontally different small letters are significantly different at (*p* < 0.05), and different capital letters within the last row and column are significantly different (*p* < 0.05), concentration (Conc.), degradation (Deg.).

Significant variations (*p* < 0.05) were demonstrated in AB1, OA, and FMB1 degradation levels, as FMB1 came in first place in its degradation levels, OA came second, while AB1 came in the last place at distances of 1.5, 2.5, and 3.5 cm, except AB1 and OA, where degradation levels were insignificantly different (*p* < 0.05) at a 3.5 cm distance (Tables 2–4). Bosch et al. [14] indicated that the variations in the structures of mycotoxins affect the efficiency of CP in mycotoxin degradation, where mycotoxins that possess long aliphatic chains (FMB1) are deteriorated swiftly in comparison with those that have a compact structure of condensed aromatic rings, as they are characterized by longer half-lives, while mycotoxins that possess mixed structures of condensed rings and aliphatic chains have intermediate degradation rates.

**Table 5.** Effect of CD at different distances and time durations on AB1 degradation levels in poultry feeds.

Treatment time (min)	Degradation (%)			
	1.5 cm	2.5 cm	3.5 cm	Time effect
0	0 ^a^	0 ^a^	0 ^a^	0 ^A^
10	46.62 ^b^	48.04 ^b^	28.92 ^c^	41.19 ^B^
20	57.73 ^d^	55.07 ^d^	39.44 ^e^	50.75 ^C^
30	65.49 ^f^	60.38 ^fd^	48.45 ^g^	58.11 ^D^
40	70.59 ^f^	66.65 ^f^	55.88 ^h^	64.37 ^E^
50	77.74 ^i^	73.68 ^i^	60.27 ^h^	70.56 ^F^
60	83.22 ^i^	80.28 ^i^	68.30 ^j^	77.26 ^G^
Distance effect	57.34 ^A^	54.87 ^A^	43.04 ^B^	

Vertically different small letters are significantly different at (*p* < 0.05), horizontally different small letters are significantly different at (*p* < 0.05), and different capital letters within the last row and column are significantly different (*p* < 0.05).

**Table 6.** Effect of CD at different distances and time durations on OA degradation levels in poultry feeds.

Treatment time (min)	Degradation (%)			
	1.5 cm	2.5 cm	3.5 cm	Time effect
0	0 ^a^	0 ^a^	0 ^a^	0 ^A^
10	49.83 ^b^	45.54 ^b^	30.17 ^c^	41.85 ^B^
20	68.93 ^d^	60.55 ^e^	40.91 ^f^	56.79 ^C^
30	73.91 ^g^	68.01 ^h^	46.74 ^i^	62.89 ^D^
40	77.38 ^g^	74.49 ^g^	57.92 ^h^	69.93 ^E^
50	82.08 ^i^	80.79 ^i^	65.37 ^j^	76.08 ^F^
60	84.21 ^i^	84.00 ^i^	71.74 ^k^	79.98 ^G^
Distance effect	62.33 ^A^	59.05 ^B^	44.69 ^C^	

Vertically different small letters are significantly different at (*p* < 0.05), horizontally different small letters are significantly different at (*p* < 0.05), and different capital letters within the last row and column are significantly different (*p* < 0.05).

**Table 7.** Effect of CD at different distances and time durations on FMB1 degradation levels in poultry feeds.

Treatment time (min)	Degradation (%)			
	1.5 cm	2.5 cm	3.5 cm	Time effect
0	0 ^a^	0 ^a^	0 ^a^	0 ^A^
10	58.63 ^b^	56.30 ^c^	36.93 ^d^	50.62 ^B^
20	75.66 ^e^	67.11 ^f^	45.27 ^g^	62.68 ^C^
30	78.37 ^h^	70.47 ^i^	56.04 ^j^	68.29 ^D^
40	80.20 ^k^	76.91 ^l^	63.84 ^m^	73.65 ^E^
50	83.67 ^n^	81.87 ^o^	72.59 ^p^	79.38 ^F^
60	84.76 ^q^	84.12 ^r^	76.18 ^s^	81.69 ^G^
Distance effect	65.9 ^A^	62.4 ^B^	50.12 ^C^	

Vertically different small letters are significantly different at (*p* < 0.05), horizontally different small letters are significantly different at (*p* < 0.05), and different capital letters within the last row and column are significantly different (*p* < 0.05).

**Figure 1.**
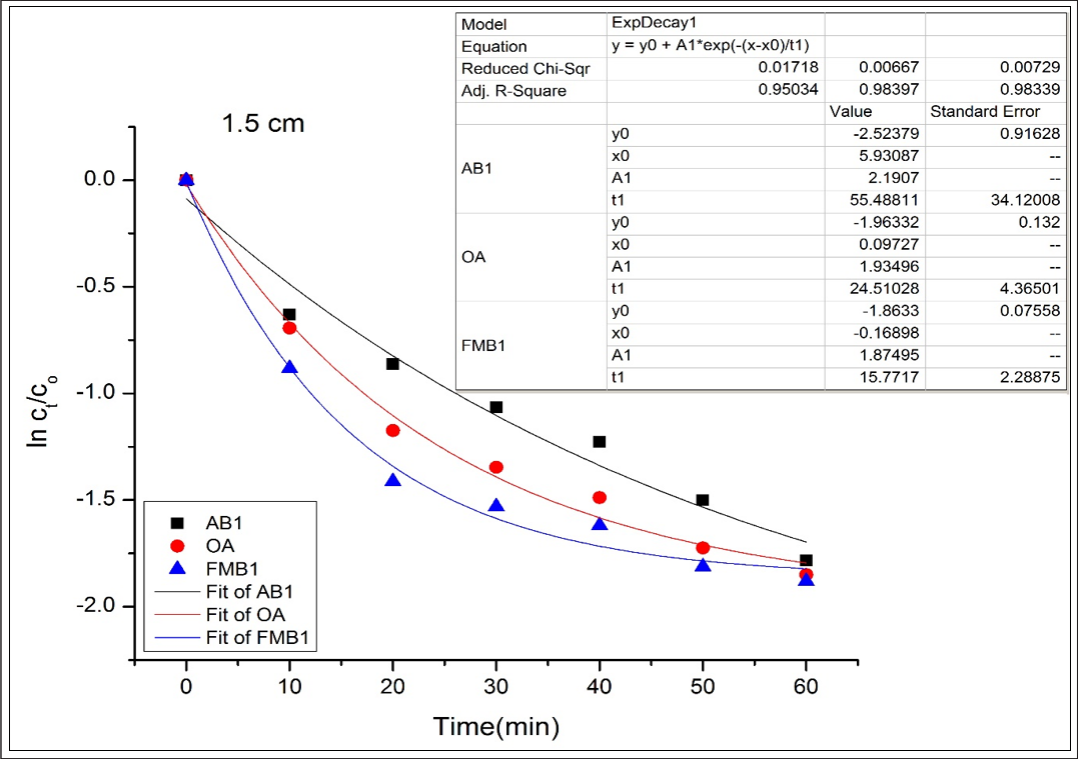
Degradation kinetics of AB1, OA, and FMB1 in poultry feeds after CD application at 1.5 cm for different time durations.

**Figure 2.**
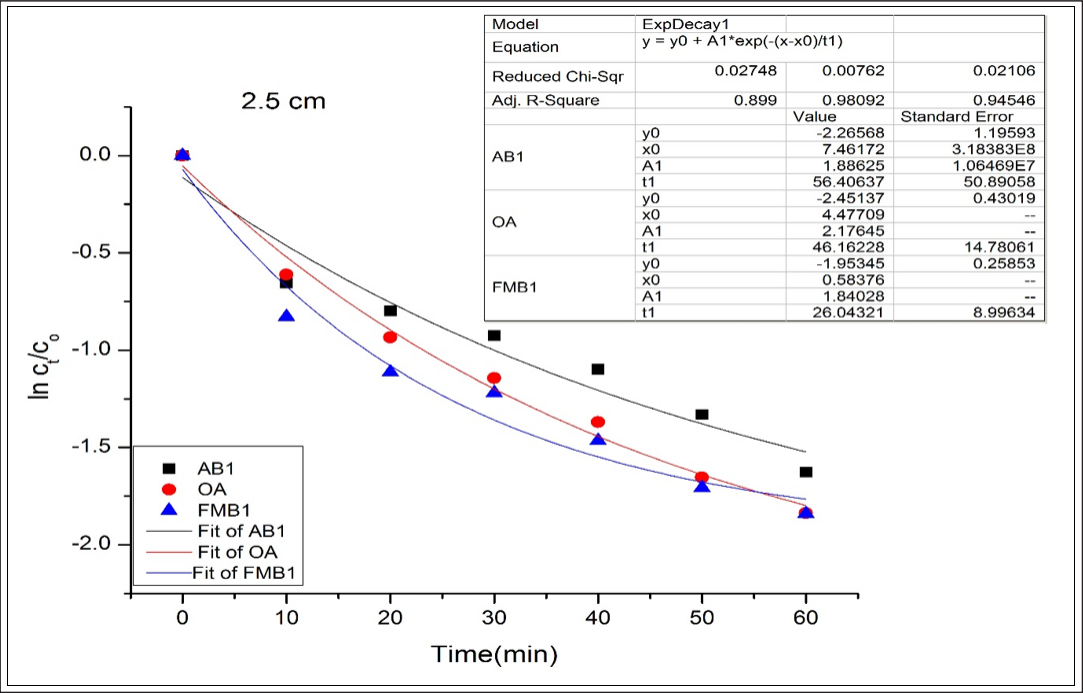
Degradation kinetics of AB1, OA, and FMB1 in poultry feeds after CD application at 2.5 cm for different time durations.

**Figure 3.**
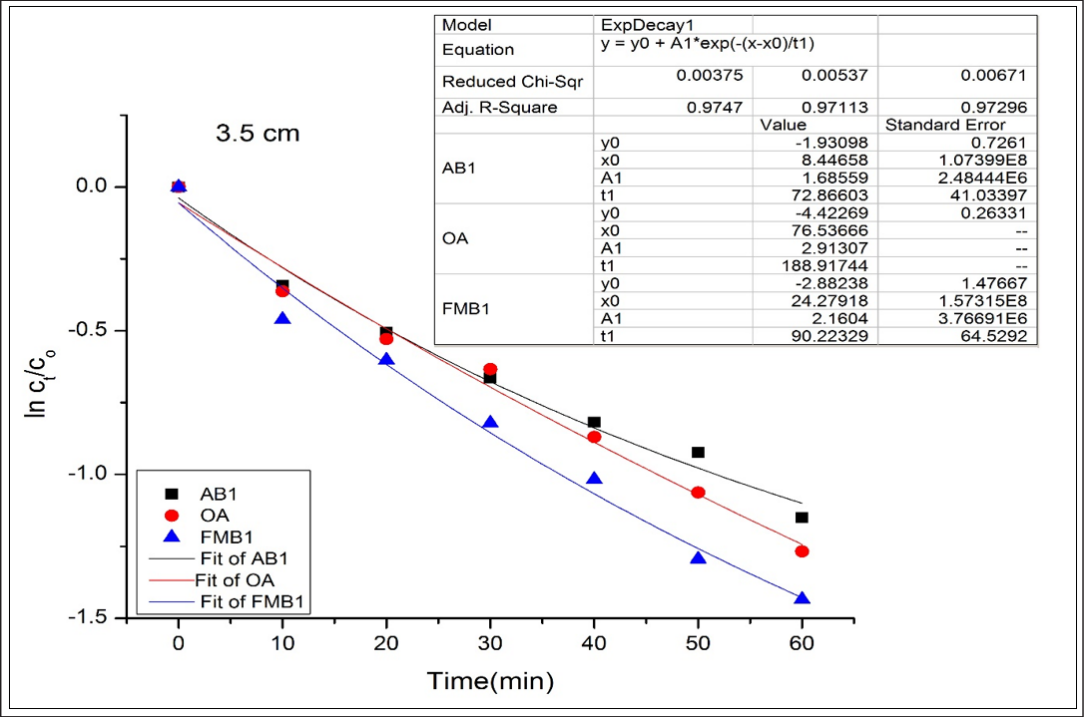
Degradation kinetics of AB1, OA, and FMB1 in poultry feeds after CD application at 3.5 cm for different time durations.

AB1, OA, and FMB1 degradation rates, following an exponential model, were well fitted (adjusted R^2^ were more than 0.94) for OA and FMB1 at the first, second, and third distances and AB1 at the first and third distances (Figs. 1–3).

Depending on HPLC analysis for confirmation of the ELISA results, results showed that AB1, OA, and FMB1 mean concentrations were lesser than those determined by ELISA with degradation levels greater than those detected by ELISA (Tables 8 and 9). Similar results were reported by Kim et al. [17].

### Impact of corona discharge on feed nutritional components and peroxide values

**Figure 4.**
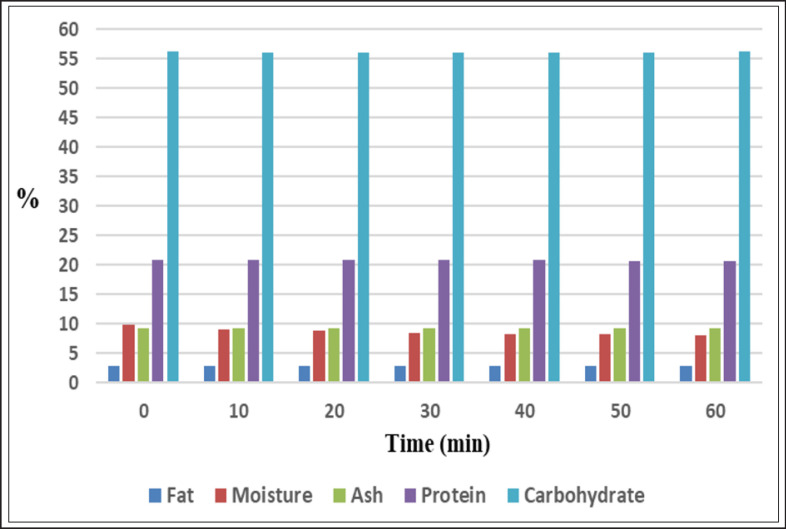
Effect of CD at 1.5 cm on the nutritional composition (%) of poultry feeds.

**Figure 5.**
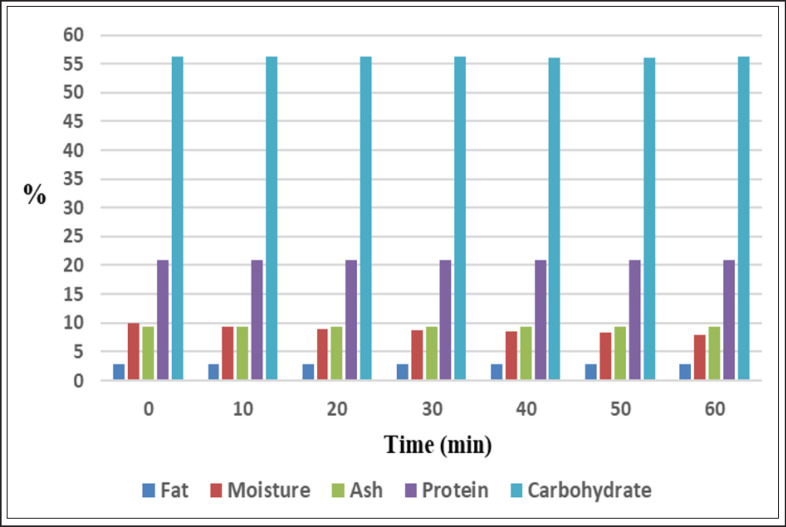
Effect of CD at 2.5 cm on the nutritional composition (%) of poultry feeds.

**Figure 6.**
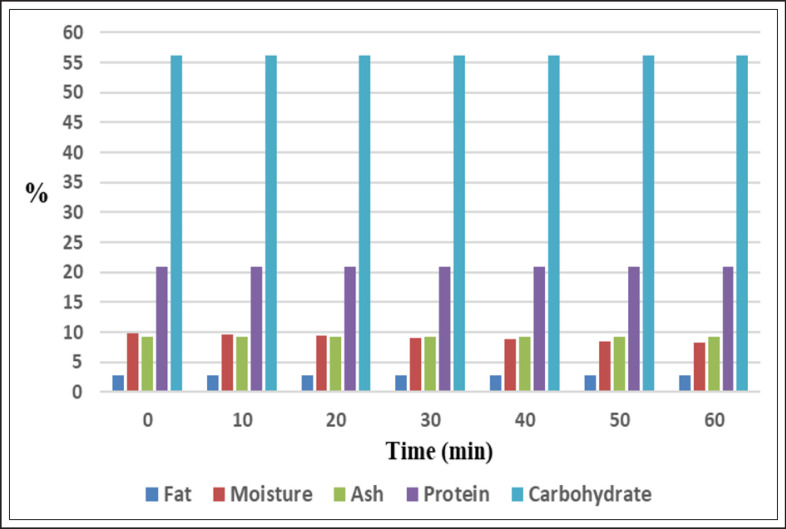
Effect of CD at 3.5 cm on the nutritional composition (%) of poultry feeds.

Analysis of nutritional components of feeds pointed out that moisture content declined significantly (*p* < 0.05) in corona discharge processed samples with processing times increment and distances reduction. Moisture decreased from 9.85% in unprocessed feed samples to 7.95%, 7.93%, and 8.33% in samples processed with CD for 60 min at the first, second, and third distances, respectively (Figs. 4–6, Table 10). Our findings aligned with the findings offered by Sarangapani et al. [30], who demonstrated that black gram moisture content decreased significantly (*p* < 0.05) with plasma power and processing period increments. Conversely, wheat moisture was not affected significantly (*p* < 0.05) after processing with dielectric barrier discharge (DBD) [31]. The extent of increment in sample temperature and the rate of the working gas flow participate significantly in decreasing sample moisture via water evaporation from processed samples. Furthermore, the processing with CP leads to the diversion of water molecules to plasma RS, resulting in a decrease in moisture [32].

At the first distance, protein content reduced significantly (*p* < 0.05) from 20.88% in unprocessed samples to a value of 20.66% after CD processing for 60 min. At the second and third distances, a non-significant increase (*p* < 0.05) was recorded in the content of protein at the first processing periods, then protein declined significantly (*p* < 0.05) after processing for 60 min to 20.82% at the second distance and insignificantly (*p* < 0.05) to 20.86% at the third distance. Moreover, the reduction in protein level at the first distance (20.78%) was significantly greater (*p *< 0.05) than the reduction recorded at the second and third distances (20.88% and 20.87%, respectively) (Figs. 4–6, Table 11). Several studies explored protein content after CP treatment, such as the study conducted by Sarangapani et al. [30], which indicated that black gram protein content increased significantly (*p* < 0.05) after exposure to plasma, attributing this increment to a reduction in moisture content. While Starek-Wojcicka et al. [33] illustrated that bread protein content was mostly not affected significantly after processing with CP. The differences in the percentages of protein may belong to the remarkable role of generated RS and energized electrons, which result in alterations in protein structural conformation and protein oxidation, causing modification in protein molecular characteristics [34].

**Table 8.** HPLC analysis of poultry feed samples showed the highest co-occurrence and highest degradation levels of AB1, OA, and FMB1 after CD treatment at 1.5 cm.

Treatment time (min)	AB1		OA		FMB1	
	Conc. (µg/kg)	Deg. (%)	Conc. (µg/kg)	Deg. (%)	Conc. (µg/kg)	Deg. (%)
0	21.84	0	149	0	6,613.5	0
50	4.04	81.52	24.87	83.31	1,048.90	84.14
60	3.28	85	19.21	87.11	904.73	86.32

Concentration (Conc.), degradation (Deg.).

**Table 9.** HPLC analysis of poultry feed samples showed the highest co-occurrence and highest degradation levels of AB1, OA, and FMB1 after CD treatment at 2.5 cm.

Treatment time (min)	AB1		OA		FMB1	
	Conc. (µg/kg)	Deg. (%)	Conc. (µg/kg)	Deg. (%)	Conc. (µg/kg)	Deg. (%)
0	21.84	0	149.00	0	6,613.50	0
50	5.16	76.39	28.44	80.91	1,169.93	82.31
60	3.39	84.47	21.87	85.32	917.95	86.12

Concentration (Conc.), degradation (Deg.).

Not-significant alterations (*p* < 0.05) in the carbohydrate content during treatment with corona discharge were registered (Figs. 4–6, Table 12). Our results were contradicted by the results obtained by Sarangapani et al. [30], which pointed out that black gram carbohydrate content differed significantly (*p* < 0.05) after plasma processing. Zare et al. [35] reported that processing by DBD affected the starch structure.

**Table 10.** Effect of CD application at different distances and time durations on the moisture content of poultry feeds.

Treatment time (min)	Moisture content (%)			
	1.5 cm	2.5 cm	3.5 cm	Time effect
0	9.85 ^a^	9.85 ^a^	9.85 ^a^	9.85 ^A^
10	9.12 ^b^	9.41 ^c^	9.72 ^d^	9.42 ^B^
20	8.74 ^e^	8.98 ^f^	9.41 ^g^	9.04 ^C^
30	8.45 ^h^	8.78 ^i^	9.05 ^j^	8.76 ^D^
40	8.22 ^k^	8.48 ^l^	8.81 ^m^	8.50 ^E^
50	8.18 ^k^	8.27 ^n^	8.56 ^o^	8.34 ^F^
60	7.95 ^p^	7.93 ^p^	8.33 ^q^	8.07 ^G^
Distance effect	8.64 ^A^	8.81 ^B^	9.10 ^C^	

Vertically different small letters are significantly different at (*p* < 0.05), horizontally different small letters are significantly different at (*p* < 0.05), and different capital letters within the last row and column are significantly different (*p* < 0.05).

The ash content of feeds was not affected significantly (*p* < 0.05) during CD processing at the different studied intervals and distances (Figs. 4–6, Table 13). Non-significant differences (*p* < 0.05) were also reported by [31] in wheat ash content after processing by DBD. Conversely, ash percentages in the black gram differed significantly (*p* < 0.05) after exposure to plasma as presented by Sarangapani et al. [30].

**Table 11.** Effect of CD application at different distances and time durations on the protein content of poultry feeds.

Treatment time (min)	Protein content (%)			
	1.5 cm	2.5 cm	3.5 cm	Time effect
0	20.88 ^a^	20.88 ^ab^	20.88 ^ab^	20.88 ^A^
10	20.84 ^ac^	20.9 ^b^	20.88 ^ab^	20.87 ^AB^
20	20.82 ^cd^	20.9 ^b^	20.89 ^b^	20.87 ^AB^
30	20.78 ^de^	20.89 ^b^	20.87 ^b^	20.85 ^BC^
40	20.76 ^ef^	20.88 ^b^	20.85 ^b^	20.83 ^C^
50	20.73 ^f^	20.86 ^bh^	20.87 ^b^	20.82 ^C^
60	20.66 ^g^	20.82 ^h^	20.86 ^hb^	20.78 ^D^
Distance effect	20.78 ^A^	20.88 ^B^	20.87 ^B^	

Vertically different small letters are significantly different at (*p* < 0.05), horizontally different small letters are significantly different at (*p* < 0.05), and different capital letters within the last row and column are significantly different (*p* < 0.05).

**Table 12.** Effect of CD application at different distances and time durations on the carbohydrate content of poultry feeds.

Treatment time (min)	Carbohydrate content (%)			
	1.5 cm	2.5 cm	3.5 cm	Time effect
0	56.16 ^a^	56.16 ^a^	56.16 ^a^	56.16 ^A^
10	56.14 ^a^	56.17 ^a^	56.15 ^a^	56.15 ^A^
20	56.15 ^a^	56.17 ^a^	56.13 ^a^	56.15 ^A^
30	56.13 ^a^	56.15 ^a^	56.15 ^a^	56.14 ^A^
40	56.13 ^a^	56.13 ^a^	56.14 ^a^	56.13 ^A^
50	56.15 ^a^	56.14 ^a^	56.14 ^a^	56.14 ^A^
60	56.17 ^a^	56.15 ^a^	56.16 ^a^	56.16 ^A^
Distance effect	56.15 ^A^	56.15 ^A^	56.15 ^A^	

Vertically different small letters are significantly different at (*p* < 0.05), horizontally different small letters are significantly different at (*p* < 0.05), and different capital letters within the last row and column are significantly different (*p* < 0.05).

Results clarified a fluctuation in the content of poultry feed fat during CD processing at the examined distances. First, fat percentages increased insignificantly (*p* < 0.05) from 2.89% in unprocessed samples to 2.92%, 2.93%, and 2.9% in processed samples at the first, second, and third distances, respectively. Thereafter, fat percentages reduced significantly (*p* < 0.05) at the first and second distances to values of 2.74% and 2.82%, respectively, and not significantly (*p* < 0.05) at the third distance to 2.86% after CD processing for 60 min. Results illustrated that the reduction in the fat percentage at the first distance (2.86%) was significantly bigger (*p* < 0.05) than the reduction recorded at the second and third distances (2.89% and 2.88%, respectively) (Figs. 4–6, Table 14).

**Table 13.** Effect of CD application at different distances and time durations on the ash content of poultry feeds.

Treatment time (min)	Ash content (%)			
	1.5 cm	2.5 cm	3.5 cm	Time effect
0	9.28 ^a^	9.28 ^a^	9.28 ^a^	9.28 ^A^
10	9.3 ^a^	9.29 ^a^	9.27 ^a^	9.29 ^A^
20	9.31 ^a^	9.3 a	9.26 ^a^	9.29 ^A^
30	9.29 ^a^	9.31 ^a^	9.27 ^a^	9.29 ^A^
40	9.27 ^a^	9.28 ^a^	9.29 ^a^	9.28 ^A^
50	9.22 ^a^	9.25 ^a^	9.28 ^a^	9.25 ^A^
60	9.15 ^a^	9.24 ^a^	9.26 ^a^	9.22 ^A^
Distance effect	9.26 ^A^	9.28 ^A^	9.27 ^A^	

Vertically different small letters are significantly different at (*p* < 0.05), horizontally different small letters are significantly different at (*p* < 0.05), and different capital letters within the last row and column are significantly different (*p* < 0.05).

**Table 14.** Effect of CD application at different distances and time durations on the fat content of poultry feeds.

Treatment time (min)	Fat content (%)			
	1.5 cm	2.5 cm	3.5 cm	Time effect
0	2.89 ^ac^	2.89 ^ac^	2.89 ^ab^	2.89 ^AB^
10	2.92 ^a^	2.91 ^ab^	2.87 ^b^	2.9 ^AB^
20	2.91 ^a^	2.93 ^a^	2.89 ^ab^	2.91 ^B^
30	2.88 ^ac^	2.92 ^a^	2.9 ^ab^	2.9 ^AB^
40	2.85 ^c^	2.89 ^ac^	2.88 ^bc^	2.87 ^A^
50	2.8 ^d^	2.85 ^cf^	2.86 ^bc^	2.84 ^C^
60	2.74 ^e^	2.82 ^f^	2.86 ^bf^	2.81 ^D^
Distance effect	2.86 ^A^	2.89 ^B^	2.88 ^B^	

Vertically different small letters are significantly different at (*p* < 0.05), horizontally different small letters are significantly different at (*p* < 0.05), and different capital letters within the last row and column are significantly different (*p* < 0.05).

Peroxide values assessment was adopted for oxidative quality appraisal of feed fats during CD processing. During the first 30 min of processing at the first distance and 50 min of processing at the second and third distances, non-significant differences (*p* < 0.05) in the peroxide values were registered. Later, a significant increment (*p* < 0.05) was recorded in PVs from 3.85 meq/kg in unprocessed samples to 4.21, 3.95, and 3.94 meq/kg after processing for 60 min at the first, second, and third distances, respectively. Moreover, PVs increment at the first distance was significantly higher (*p* < 0.05) than those estimated at the second and third distances (Table 15).

**Table 15.** Effect of CD application at different distances and time durations on the peroxide values of poultry feed fats.

Treatment time (min)	Peroxide values (meq/kg)			
	1.5 cm	2.5 cm	3.5 cm	Time effect
0	3.85 ^a^	3.85 ^a^	3.85 ^a^	3.85 ^A^
10	3.84 ^a^	3.86 ^a^	3.88 ^a^	3.86 ^AB^
20	3.87 ^a^	3.85 ^a^	3.86 ^a^	3.86 ^AB^
30	3.87 ^a^	3.87 ^a^	3.84 ^a^	3.86 ^AB^
40	3.93 ^b^	3.88 ^a^	3.85 ^a^	3.89 ^B^
50	4.05 ^c^	3.89 ^a^	3.88 ^a^	3.94 ^C^
60	4.21 ^d^	3.95 ^b^	3.94 ^b^	4.03 ^D^
Distance effect	3.95 ^A^	3.88 ^B^	3.87 ^B^	

Vertically different small letters are significantly different at (*p* < 0.05), horizontally different small letters are significantly different at (*p* < 0.05), and different capital letters within the last row and column are significantly different (*p* < 0.05).

Results demonstrated a significant increment (*p* < 0.05) in peroxide values after processing by CD, but values were less than 5 meq/kg, suggesting that fats were still of excellent quality [36]. Other researchers documented the influence of CP on the fat content and peroxide values of the fats. Not-significant variations (*p *< 0.05) were reported by [31] in the wheat fat content after DBD processing. Significant variations (*p* < 0.05) were registered by Sarangapani et al. [30] in the black gram fat content, which fluctuated between decrease and increase after subjecting it to plasma. Gebremical et al. [37] reported an increase in the peroxide values after peanut exposure to DBD. Finally, the fat content of the bread reduced slightly (about 28%) after exposure to CP [33]. These changes in fat content are attributed to the remarkable role of the RS generated by plasma in lipid oxidation [38].

## Conclusion

It was shown in the study that CD is a promising non-thermal method for getting rid of AB1, OA, and FMB1 from poultry feeds. Ozone was the most prominent RS generated. Mycotoxin degradation increased with increasing the exposure time and decreasing the distances. According to levels of mycotoxins estimated in the current research, results illustrated that CD processing for 10 min was sufficient to reach the AB1 legal limit at the three examined distances and the OA legal limit at the first and second distances. Mycotoxins could be arranged according to their degradation levels in the following order: FMB1 > OA > AB1. Analysis of feed components revealed a significant reduction (*p* < 0.05) in moisture, protein, and fat contents. The fats were still fresh depending on PV's determination. The CD treatment did not adversely affect the carbohydrate and ash contents.

## List of abbreviations

AB1: aflatoxin B1; CD: corona discharge; cm: centimeter; CP: cold plasma; DBD: dielectric barrier discharge; DC: direct current; EC: European Commission; ELISA: Enzyme-Linked Immunosorbent Assay; FMB1: fumonisin B1; HPLC: High-Performance Liquid Chromatography; Hz: hertz; kg: kilogram; kV: kilovolt; M: molarity; ml: milliliters; min: minutes; nm: nanometer OA: ochratoxin A; ppm: parts per million; ppb: parts per billion; PV: peroxide value; ROS: reactive oxygen species; RNS: reactive nitrogen species; μw: microwatt; v: volt.

## References

[1] Awuchi CG, Ondari EN, Ogbonna CU, Upadhyay AK, Baran K, Okpala CO (2021). Mycotoxins affecting animals, foods, humans, and plants: types, occurrence, toxicities, action mechanisms, prevention, and detoxification strategies-a revisit. Foods.

[2] Jawad BJ, ALwan MJ (2020). Influence of mycotoxins on immune responses against *Salmonella typhimurium* infection of broiler chickens. Iraqi J Agric Sci.

[3] Guo J, Yan W, Tang J, Jin X, Xue H, Wang T (2022). Dietary phillygenin supplementation ameliorates aflatoxin B1-induced oxidative stress, inflammation, and apoptosis in chicken liver. Ecotoxicol Environ Saf.

[4] Dey DK, Kang JI, Bajpai VK, Kim K, Lee H, Sonwal S (2023). Mycotoxins in food and feed: toxicity, preventive challenges, and advanced detection techniques for associated diseases. Crit Rev Food Sci Nutr.

[5] El-Sayed RA, Jebur AB, Kang W, El-Demerdash FM (2022). An overview on the major mycotoxins in food products: characteristics, toxicity, and analysis. J Future Foods.

[6] Minati MH, Mohammed-Ameen MK (2023). First report of three kinds of mycotoxins deoxynivalenol, nivalenol and fumonisin B2 in seeds of seven wheat cultivars in Iraq. Iraqi J Vet Med.

[7] Mohamed AM, Al-Shamary EI (2022). Isolation and identification of aflatoxin B1 producing fungi from stored wheat in some silos of Baghdad. Iraqi J Agric Sci.

[8] Mehtab U, Tahir M, Abbas R, Abbas A, Hussain K, Siddiqui F (2021). Ochratoxin A occurrence, its pathological effects on poultry health and decontamination approaches. J Hellenic Vet Med Soc.

[9] Nava-Ramirez MJ, Maguey-Gonzalez JA, Gomez-Rosales S, Hernandez-Ramirez JO, Latorre JD, Du X (2024). Efficacy of powdered alfalfa leaves to ameliorate the toxic effects of aflatoxin B1 in turkey poults. Mycotoxin Res.

[10] Ferreira CD, Lang GH, Lindemann I, Timm N, Hoffmann JF, Ziegler V (2021). Postharvest UV-C irradiation for fungal control and reduction of mycotoxins in brown, black, and red rice during long-term storage. Food Chem.

[11] Marshall H, Meneely JP, Quinn B, Zhao Y, Bourke P, Gilmore BF (2020). Novel decontamination approaches and their potential application for post-harvest aflatoxin control. Trends Food Sci Technol.

[12] Allai FM, Azad AA, Mir NA, Gul K (2023). Recent advances in non-thermal processing technologies for enhancing shelf life and improving food safety. Appl Food Res.

[13] Yousefi M, Mohammadi MA, Khajavi MZ, Ehsan A, Scholtz V (2021). Application of novel non-thermal physical technologies to degrade mycotoxins. J Fungi.

[14] Bosch L, Pfohl K, Avramidis G, Wieneke S, Viol W, Karlovsky P (2017). Plasma-based degradation of mycotoxins produced by *Fusarium*, *Aspergillus* and *Alternaria* species. Toxins (Basel).

[15] Shi H, Cooper B, Stroshine RL, Ileleji KE, Keener KM (2017). Structures of degradation products and degradation pathways of aflatoxin B1 by high-voltage atmospheric cold plasma (HVACP) treatment. J Agric Food Chem.

[16] Puligundla P, Lee T, Mok C (2019). Effect of corona discharge plasma jet treatment on the degradation of aflatoxin B1 on glass slides and in spiked food commodities. Lebensm Wiss Technol.

[17] Kim EK, Shon DH, Yoo JY, Ryu D, Lee C, Kim YB (2001). Natural occurrence of aflatoxins in Korean meju. Food Addit Contam.

[18] Nesheim S, Stack ME, Trucksess MW, Eppley RM, Krogh P (1992). Rapid solvent-efficient method for liquid chromatographic determination of ochratoxin A in corn, barley, and kidney: Collaborative study. J AOAC Int.

[19] Shephard GS, Sydenham EW, Thiel PG, Gelderblom WC (1990). Quantitative determination of fumonisins B1 and B2 by high-performance liquid chromatography with fluorescence detection. J Liq Chromatogr.

[20] Latimer GW, AOAC (Association of Official Analytical Chemists) (1996). Moisture in animal feed, method 930.15. Official Methods of Analysis of AOAC International.

[21] DuBois M, Gilles KA, Hamilton JK, Rebers PA, Smith F (1956). Colorimetric method for determination of sugars and related substances. Anal Chem.

[22] Latimer GW, AOAC (Association of Official Analytical Chemists) (2006). Official Methods of Analysis of AOAC International.

[23] Latimer GW, AOAC (Association of Official Analytical Chemists) (1990). Protein (crude) determination in animal feed: copper catalyst kjeldahl method 984.13. Official Methods of Analysis of AOAC International.

[24] Thiex N, Novotny L, Crawford A (2012). Determination of ash in animal feed: AOAC official method 942.05 revisited. J AOAC Int.

[25] Horwitz W, AOAC (Association of Official Analytical Chemists) (2000). AOAC official method 965.33: Peroxide value. Official method of analysis of AOAC International.

[26] Steel RG, Torrie JH (1960). Principles and procedures of statistics. (With special reference to the biological sciences).

[27] Chang EH, Bae YS, Shin IS, Choi HJ, Lee JH, Choi JW (2018). Microbial decontamination of onion by corona discharge air plasma during cold storage. J Food Quality.

[28] EC (European Commission) (2011). Commission Regulation (EU) 2011/574 of 16 June 2011 Amending Annex I to Directive 2002/32/EC of the European Parliament and of the Council as regards maximum levels for nitrite, melamine, ambrosia spp. and carry-over of certain coccidiostats and histomonostats and consolidating. O J L.

[29] EC (European Commission) (2006). Commission Recommendation 2006/576/EC of 17 August 2006 on the presence of deoxynivalenol, zearalenone, ochratoxin A, T-2 and HT-2 and fumonisins in products intended for animal feeding. O J L.

[30] Sarangapani C, Devi RY, Thirumdas R, Trimukhe AM, Deshmukh RR, Annapure US (2017). Physico-chemical properties of low-pressure plasma treated black gram. LWT - Food Sci Tech.

[31] Mohammadi S, Imani S, Dorranian D, Tirgari S, Shojaee M (2015). The effect of non-thermal plasma to control of stored product pests and changes in some characters of wheat materials. J Biodivers Environ Sci.

[32] Shirani K, Shahidi F, Mortazavi SA (2020). Investigation of decontamination effect of argon cold plasma on physicochemical and sensory properties of almond slices. Int J Food Microbiol.

[33] Starek-Wojcicka A, Rozylo R, Niedzwiedz I, Kwiatkowski M, Terebun P, Polak-Berecka M (2022). Pilot study on the use of cold atmospheric plasma for preservation of bread. Sci Rep.

[34] Olatunde OO, Hewage A, Dissanayake T, Aluko RE, Karaca AC, Shang N (2023). Cold atmospheric plasma-induced protein modification: novel nonthermal processing technology to improve protein quality, functionality, and allergenicity reduction. Compr Rev Food Sci Food Saf.

[35] Zare L, Mollakhalili-Meybodi N, Fallahzadeh H, Arab M (2022). Effect of atmospheric pressure cold plasma (ACP) treatment on the technological characteristics of quinoa flour. LWT – Food Sci Tech.

[36] Wealleans AL, Bierinckx K, Witters E, di Benedetto M, Wiseman J (2021). Assessment of the quality, oxidative status and dietary energy value of lipids used in non-ruminant animal nutrition. J Sci Food Agric.

[37] Gebremical GG, Emire SA, Berhanu T (2019). Effects of multihollow surface dielectric barrier discharge plasma on chemical and antioxidant properties of peanut. J Food Qual.

[38] Wang X, Wang J, Wang Z, Yan W, Zhuang H, Zhang J (2023). Impact of dielectric barrier discharge cold plasma on the lipid oxidation, color stability, and protein structures of myoglobin-added washed pork muscle. Front Nutr.

